# Structural and functional studies on *Pseudomonas aeruginosa* DspI: implications for its role in DSF biosynthesis

**DOI:** 10.1038/s41598-018-22300-1

**Published:** 2018-03-02

**Authors:** Li Liu, Tao Li, Xing-Jun Cheng, Cui-Ting Peng, Chang-Cheng Li, Li-Hui He, Si-Min Ju, Ning-Yu Wang, Ting-Hong Ye, Mao Lian, Qing-Jie Xiao, Ying-Jie Song, Yi-Bo Zhu, Luo-Ting Yu, Zhen-Ling Wang, Rui Bao

**Affiliations:** 1 Center of Infectious Diseases, West China Hospital, Sichuan University, Chengdu, China; 2 Department of Dermatology, Southwest Medical University, affiliated hospital, Luzhou, China; 3 School of Life Science and Engineering, Southwest Jiaotong University, Chengdu, China

## Abstract

DspI, a putative enoyl-coenzyme A (CoA) hydratase/isomerase, was proposed to be involved in the synthesis of *cis*-2-decenoic acid (CDA), a quorum sensing (QS) signal molecule in the pathogen *Pseudomonas aeruginosa* (*P*. *aeruginosa*). The present study provided a structural basis for the dehydration reaction mechanism of DspI during CDA synthesis. Structural analysis reveals that Glu126, Glu146, Cys127, Cys131 and Cys154 are important for its enzymatic function. Moreover, we show that the deletion of *dspI* results in a remarkable decreased in the pyoverdine production, flagella-dependent swarming motility, and biofilm dispersion as well as attenuated virulence in *P*. *aeruginosa* PA14. This study thus unravels the mechanism of DspI in diffusible signal factor (DSF) CDA biosynthesis, providing vital information for developing inhibitors that interfere with DSF associated pathogenicity in *P*. *aeruginosa*.

## Introduction

*Pseudomona aeruginosa* (*P*. *aeruginosa*), as a common nosocomial gram-negative pathogenic bacterium, can inhabit a wide variety of ecological niches and infect diverse hosts. Owing to its intrinsic multi-drug resistance, *P*. *aeruginosa* is one of the leading causes of healthcare-associated infections, calling for effective treatments or agents with novel anti-infective mechanisms. In response to cell density or confinement to niches, *P*. *aeruginosa* adopts various signal molecules to mediate virulence factors biosynthesis and/or biofilm formation. Therefore, inhibiting these signaling pathways represents attractive strategies for developing novel therapeutics against *P*. *aeruginosa* infection^1–4^.

In many pathogenic bacteria, quorum-sensing (QS) signaling is an important regulatory switch contributing to bacteria virulence and persistence^5^. By producing and releasing hormone-like chemical signal molecules involved in bacterial QS system, bacteria can communicate intercellularly to regulate a variety of physiological activities, such as motility, virulence, antibiotic production and biofilm dispersion. In the past few years, the diffusible signal factor (DSF) family has been disclosed as a new type of QS system signal that is common in gram-negative bacterial pathogens^6,7^. The first identified DSF family molecule *cis-*11-methyl-2-dodecenoic acid, was found in the plant pathogen *Xanthomonas campestris* pv. *campestris* (*X*.*cc*)^8^, which regulates many biological functions including cell growth, biofilm dispersion and virulence^9^. *Cis*-2-decenoic acid (CDA) is a new member of the DSF family that was found in *P*. *aeruginosa* and functions as an auto-inducer for biofilm dispersion^10,11^. Additionally, as an inter-kingdom signaling molecule, CDA also regulates the biofilm formation and dispersion in a number of other pathogens^12–15^.

So far, multiple DSF family molecules have been detected in various pathogens^7^. A particular group of particular enoyl-coenzyme A (CoA) hydratase/isomerases includes RpfF from *X*.*cc*, which appears to be the key enzyme in DSF biosynthesis^7,16–21^. In *P*. *aeruginosa*, the gene *PA0745* encodes a putative crotonase, named *dspI*, which has been shown to be responsible for the CDA biosynthesis^10^. Meanwhile, *dspI* has been verified to be required for *P*. *aeruginosa* virulence in the *Caenorhabditis elegans* (*C*. *elegans*) infection-based killing model for *P*. *aeruginosa* virulence factor screening, which further suggested its role as a potential drug target^22^. However, the detailed molecular mechanism of CDA biosynthesis mediated by DspI and the relationship between DspI and *P*. *aeruginosa* pathogenicity remains unclear.

In this study, we examined the role of DspI in *P*. *aeruginosa* pathogenicity via its regulation on the production of the virulence factor pyocyanin producing, swarming motility and biofilm dispersion. The structural studies confirmed the catalytic features of DspI as an enoyl-coenzyme A (CoA) hydratase that catalyzes the dehydration of 3-hydroxydecanoyl-CoA during CDA synthesis. Moreover, structural analysis combined with mutagenesis and the chronic airway infection mouse model allowed us to identify critical residues for DspI function. The result sheds light on the mechanism of how DspI modulates CDA biosynthesis and its impacts on *P*. *aeruginosa* infection, providing the starting point for structure-based drug development targeting QS-associated virulence.

## Results

### DspI resembles a typical crotonase fold and assembles as a homotrimer

Recombinant DspI with a C-terminal his-tagged was purified and crystalized. The proteins were crystallized in two different space groups. The P3_1_ form has six molecules and the P6_3_22 form has only one molecule per asymmetric units. The atomic coordinates from the two space groups were refined at a resolution of 2.10 Å and 2.25 Å. The crystallographic and refinement statistics are shown in Table 1. In both crystal forms, the first eight residues have not been modeled because of the poor density in this region. The C-terminal segment (residue 252–272) is further missing in the P6_3_22 form. Thus, the structure of the P3_1_ form is used for most of the descriptions in this study, unless otherwise specified.

**Table 1:** Statistics on the qualities of diffraction data and model refinement of DspI.

Data collection		
Space group	P 3_1_	P 6_3_ 2 2
**Cell dimensions**		
*a*, *b*, *c* (Å)	83.309 83.309 207.547	125.262 125.262 72.651
α, β, γ (°)	90 90 120	90 90 120
Wavelength	0.97022	0.97776
Resolution (Å)	40.00–2.10(2.18–2.10)^a^	30–2.15(2.23–2.15)
Rsym	0.074(0.466)	0.157(0.621)
I/σI	15.44(1.9)	19(3.25)
Completeness (%)	96.2(92.1)	100(99.9)
Redundancy	5.0(3.0)	20.5(12.9)
**Refinement**		
Resolution (Å)	40.00–2.10(2.14–2.10)	28.7–2.25(2.31–2.25)
No. of reflections	90298(4323)	16394(1330)
Rwork/Rfree^b^	0.2271/0.2762 (0.3250/0.3947)	0.2302/0.2651 (0.3446/0.3508)
**No**. **of atoms**		
Protein	12130	1864
Ligand/ion	64	19
Water	208	95
**B-factors**(Å^2^)	51.85	42.98
Protein	52.23	42.89
Ligand/ion	32.58	66.56
Water	34.22	39.94
**r**.**m**.**s**.**d**.		
Bond lengths (Å)	0.012	0.015
Bond angles (°)	1.37	1.3
Ramachandran plot favored/allowed	98.6/1.4	96.7/3.3

^a^Numbers in parentheses are statistics of the outer shell. ^b^5% of total reflections were set aside for the Rfree calculation.

**Figure 1:**
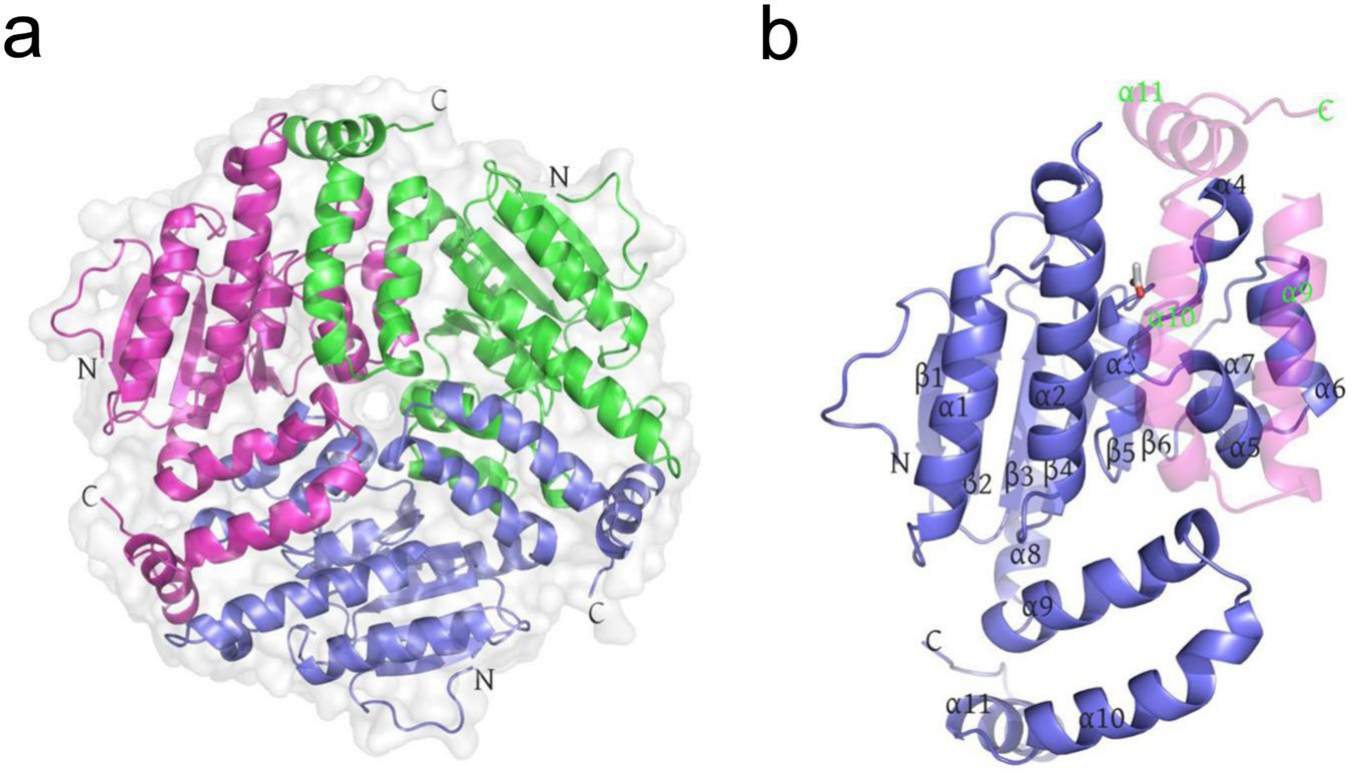
DspI resembles a typical crotonase fold and assembles as a homotrimer. (**a**) Cartoon representation of the DspI trimer. Each subunit is shown in a different color. (**b**) Cartoon style of the DspI monomer. The secondary structure elements are labeled and the C-domain from the neighbor subunit is shown as a transparent cartoon.

Hexamer organizations could be generated by applying the symmetry operations in both crystal forms. The hexamer is a dimer of two stacked trimers and each subunit possesses the canonical crotonase fold. The trimeric oligomerization of DspI is shown in Fig. 1a. Three subunits associated with each other tightly through a complementary interaction, which resulted in an average interface area of 2012.5 Å^2^ and 1711.8 Å^2^ in the P3_1_ form and P6_3_22 form, respectively.

DspI is a α/β protein composed of six perpendicular antiparallel β-strands surrounded by eleven α-helices (Fig. 1b). It can be divided into two domains: the N-terminal spiral domain (α1–α8 and β1–β6) and the C-terminal trimerization domain (α9-end). The helix-helix contacts between the N-terminal extension together with the trimerization domain of the neighboring monomer stabilize the homo-trimeric disk assembly (Fig. 1a). This head-to-tail swapping pattern is basically conserved in many crotonase superfamily (CS) members except for those with different C-terminal α-helix orientations^23^. In DspI, the C-terminal residue 252–272 (average B factor 73.54 Å^2^) is more flexible than the remainder of the trimerization domain (average B factor 41.71 Å^2^). It protrudes away from its own subunit and covers the neighboring monomer within the same trimer. This type of intra-trimer interactions has been proposed to be a common feature of enoyl-CoA hydratase^23^.

### Identification of critical residues in the active-site pocket

**Figure 2:**
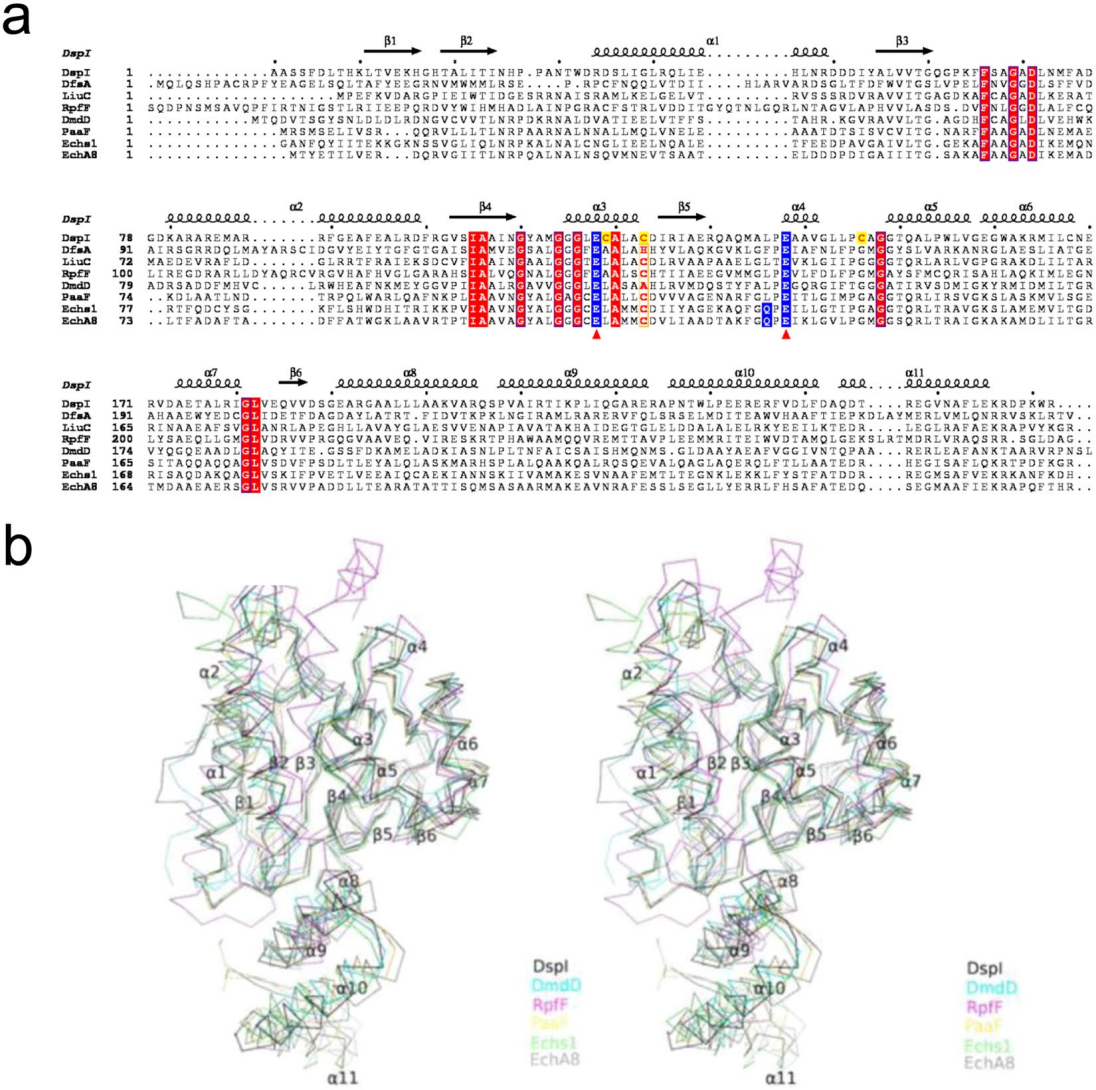
Sequence alignment of the ECH/ECI enzymes from different bacterial species. (**a**) The secondary structure elements of DspI are indicated above the sequences. The catalytic Glu residues are colored blue and the Cys127, Cys131, and Cys154 from DspI that are highlighted in yellow that are the binding sites of CDA-CoA with DspI. (**b**) Superposition of the DspI and ECH/ECI enzymes.

Previous structural studies have characterized several enoyl-CoA hydratase/isomerase (ECH/ECI) including DsfA (PDB: 5FUS) from *Burkholderia cenocepacia*^24^, LiuC (PDB: 5JBX) from *Myxococcus xanthus*^25^, DmdD (PDB: 4IZB) from *Ruegeria pomeroyi*^26^, Echs1 (PDB: 1DCI) from *Rattus norvegicus*^27^, PaaF (PDB: 4FZW) from *Escherichia coli*^28^, RpfF (PDB: 3M6N) from *Xcc* and EchA8 (PDB: 3Q0G) from *Mycobacterium tuberculosis*^9,29^ (Fig. 2a). Despite low sequence identities (18.9–35.8%) between these ECH/ECI enzymes and DspI, they shared similar secondary structure assignments with a common N-terminal core domain (RMSD 0.684–1.618 Å^2^) but differed in the C-terminal trimerization domain (RMSD 1.164–1.923 Å^2^) (Fig. 2b). In DspI, a surface region including loops connecting β2-α1, β3-α2 and β4-α3 was relatively conserved for the binding of the CoA moiety binding (Fig. 2a). The active site following this CoA binding site possesses two well-conserved acidic residues, Glu126 and Glu146. These two Glu residues were critical for catalysis and have been concluded as one of the structural features to differentiate the ECH hydration or bifunctional ECH/ECI activity from the monofunctional ECI or DCI (dienoyl-CoA isomerase) activity^29,30^.

**Figure 3:**
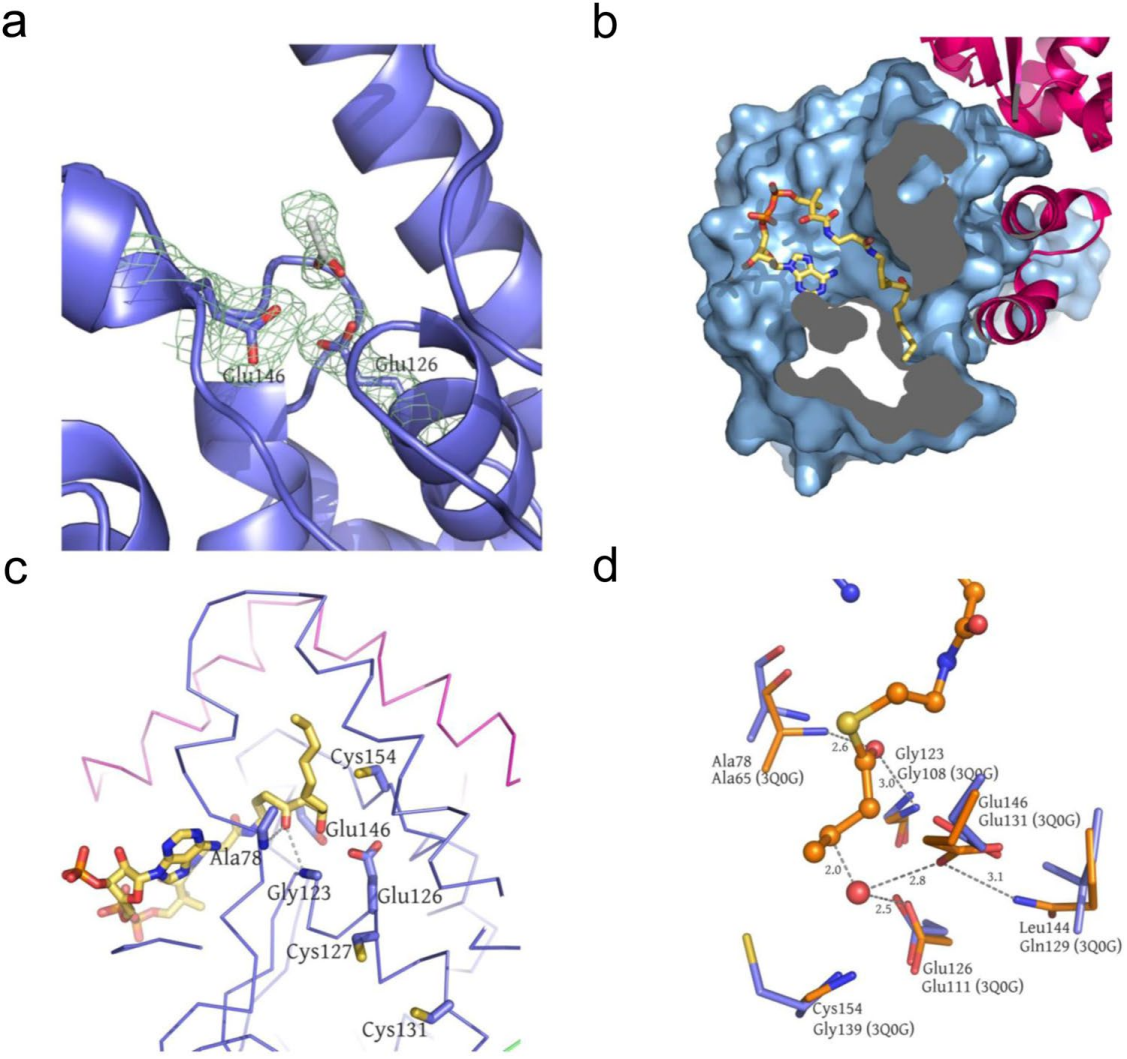
(**a**) Close-up view of the catalytic center in the P6_3_22 crystal form, with an acetic acid to be built in. (**b**) Dock 3-hydroxydecanoyl-CoA into the DspI substrate binding pocket using AutoDock suite. The hydrophobic binding pocket of the acyl portion of the substrates is shown in the cutaway view and ribbon style (**c**). (**d**) Close-up view of the active-site pocket of DspI (blue) superimposed on the ECH/ECI 3Q0G (orange). Critical residues are shown in the stick style.

Although the crystal soaking and co-crystallization did not generate the substrate (or analogue) that bound the DspI structure, the 2Fo-Fc difference map around the active site in the P6_3_22 form allowed us to model an acetic acid molecule in it (Fig. 3a). The hydroxyl group from the acetic acid points toward residue Glu126 and Glu146, implicating the possible binding pattern for the 3′ hydroxyl group of the CDA-CoA precursor 3-hydroxydecanoyl-CoA. Since the U-shape binding pattern of the CoA portion is highly conserved in most of the CS members^23^ and the reaction equation has been proposed in Amari *et al*.^19^, we generated a (R)3-hydroxydecanoyl-CoA model based on the butyryl-CoA ligand from the EchA8 structure (PDB: 3Q0G), and docked it into the DspI substrate binding pocket using the AutoDock suite^31^ (Fig. 3b). Cavity analysis reveals that the binding tunnel passes through the active site and the carbon chain tail is guided towards a hydrophobic bottom formed by a flexible loop (residues 79–87), which has larger B-factor (105.7 Å^2^) than the average (42.89 Å^2^). Additionally, the α10–α11 from the neighboring subunit further stabilizes the substrate binding through the hydrophobic interactions. Close inspection of the modeled DspI-substrate complex shows a conserved oxyanion hole (OAH) formed by the main chain NH atoms of Ala78 and Gly123, which serves as the primary platform stabilizing enolate/oxyanion intermediates during CS catalyzing^23^ (Fig. 3c). Notably, in addition to the two catalytic residues Glu126 and Glu146, three nearby cysteine residues nearby (Cys127, Cys131 and Cys154) were identified and predicted to be important for DspI activity. Especially the Cys154, which occupied the third acidic residue position involved in some ECH/ECI catalyzing^29^ and may contribute the thiol group as a nucleophile during enzymatic reactions (Fig. 3d).

### *dspI* deletion reduced *P*. *aeruginosa* swarming motility via impaired flagellar assembly

**Figure 4:**
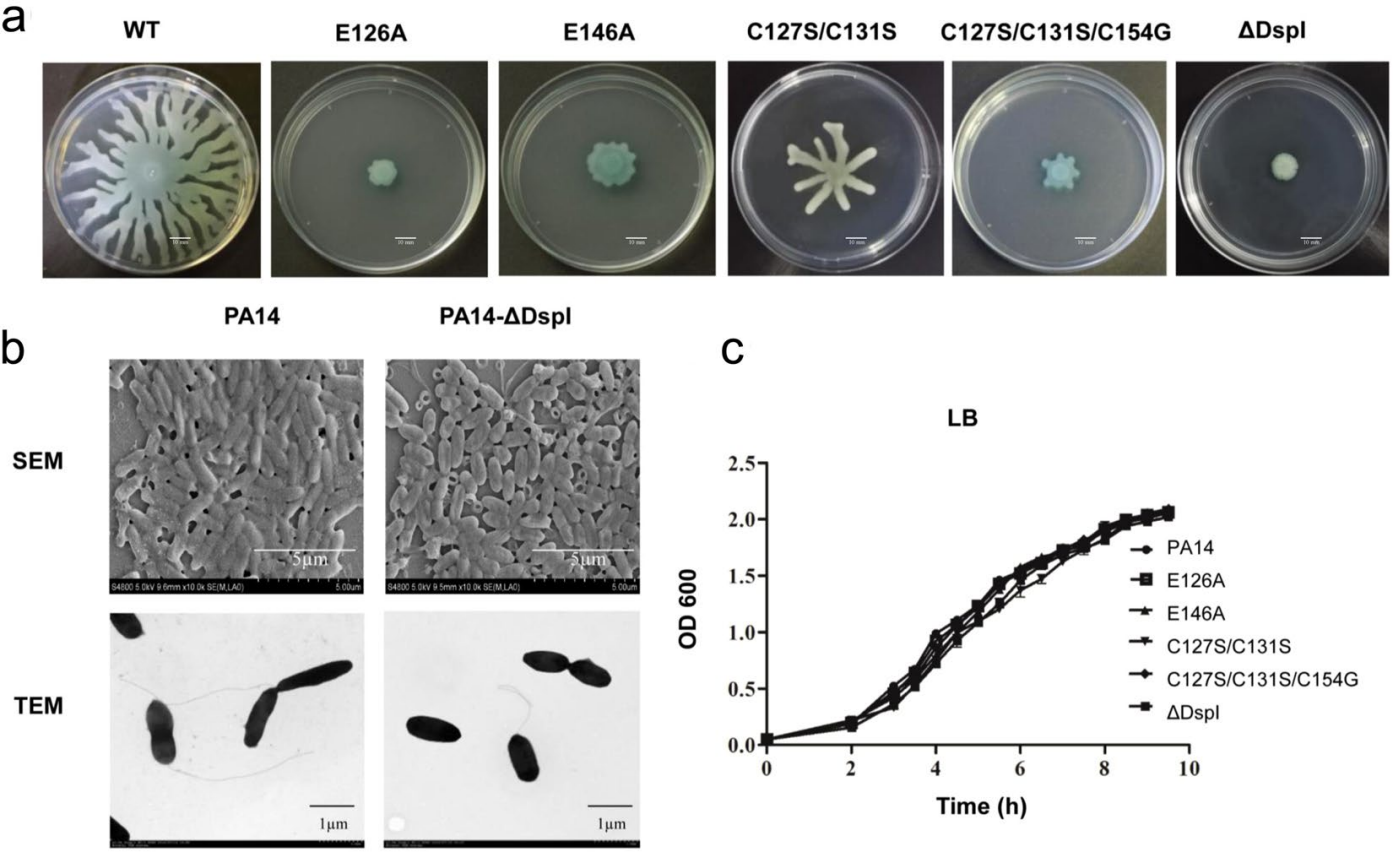
Swarming motility and morphology of *P*. *aeruginosa* PA14 as well as strains carrying mutations. (**a**) Representative images of swarming motility. Reduced swarm coverage includes reduced tendrils (PA14 C127S/C131S) or tendril-less growth of the colony (PA14 E126A, E146A, C127S/C131S/C154G, ΔDspI). (**b**) Morphology of *P*. *aeruginosa* swarm cells by SEM (Upper) and TEM (Lower) of bacteria taken directly from swarm plates strains: edge cells of PA14 (left) and PA14-ΔDspI colony (right). (**c**) The measurement of the growth curve of wild-type *P*. *aeruginosa* PA14 and mutations at 37 °C in LB medium. Mutations exhibited no growth defect relative to *P*. *aeruginosa* PA14. Measurements were performed three times.

The previous study has reported that *dspI* deletion in PA14 inhibited CDA biosynthesis, leading to less biofilm dispersion but greater biomass and thickness in biofilm production than the wild-type^10^. The DspI also regulates the swarming motility and biofilm formation^22^. To confirm the function of DspI in *P*. *aeruginosa* motility, the in-frame deletion mutant PA14 *ΔdspI* was constructed and then the swimming, twitching and swarming motilities were examined on 0.3%, 1%, and 0.5% (wt/vol) agar plates, respectively. PA14 *ΔdspI* exhibited only minor differences from the wild-type PA14 strains (WT) in swimming and twitching motility (data not shown). In contrast, the WT dendritic swarming pattern was not detected in PA14 *ΔdspI*. The swarming motility had varying degrees of reduction was observed in different *dspI* mutants (Fig. 4a, S1). Furthermore, the swarming-defective phenotype of the PA14 *ΔdspI* could be reverted by adding exogenous CDA (Fig. S2). These results, in accordance with the previously published studies^22^, confirmed a modulation role of DspI in the swarming motility via the CDA production.

It was shown that the swarming motility of *P*. *aeruginosa* is flagella-dependent^32^. Thus, we further examined the morphology difference between ΔdspI and the WT strains by SEM and TEM. As Fig. 4b and Fig. S3 shows, the *ΔdspI* mutant was slightly shorter than WT cells. The elongated WT cells often possessed one or more polar flagella while a single or no polar flagellum was observed in the *ΔdspI* strain. Meanwhile, to investigate whether the swarming-deficient phenotype observed in the *ΔdspI* strain was related to differences in growth behavior, the OD_600_ values of the *ΔdspI* and the WT were monitored at 37 °C in LB medium. As shown in Fig. 4c and Fig. S4, the PA14 *ΔdspI* strain exhibited no growth difference from the WT strains. Thus, the *dspI* knockout in *P*. aeruginosa PA14 impaired flagella-dependent swarming motility but had no significant effect on growth.

### DspI positively regulates bacterial virulence and dispersion

**Figure 5:**
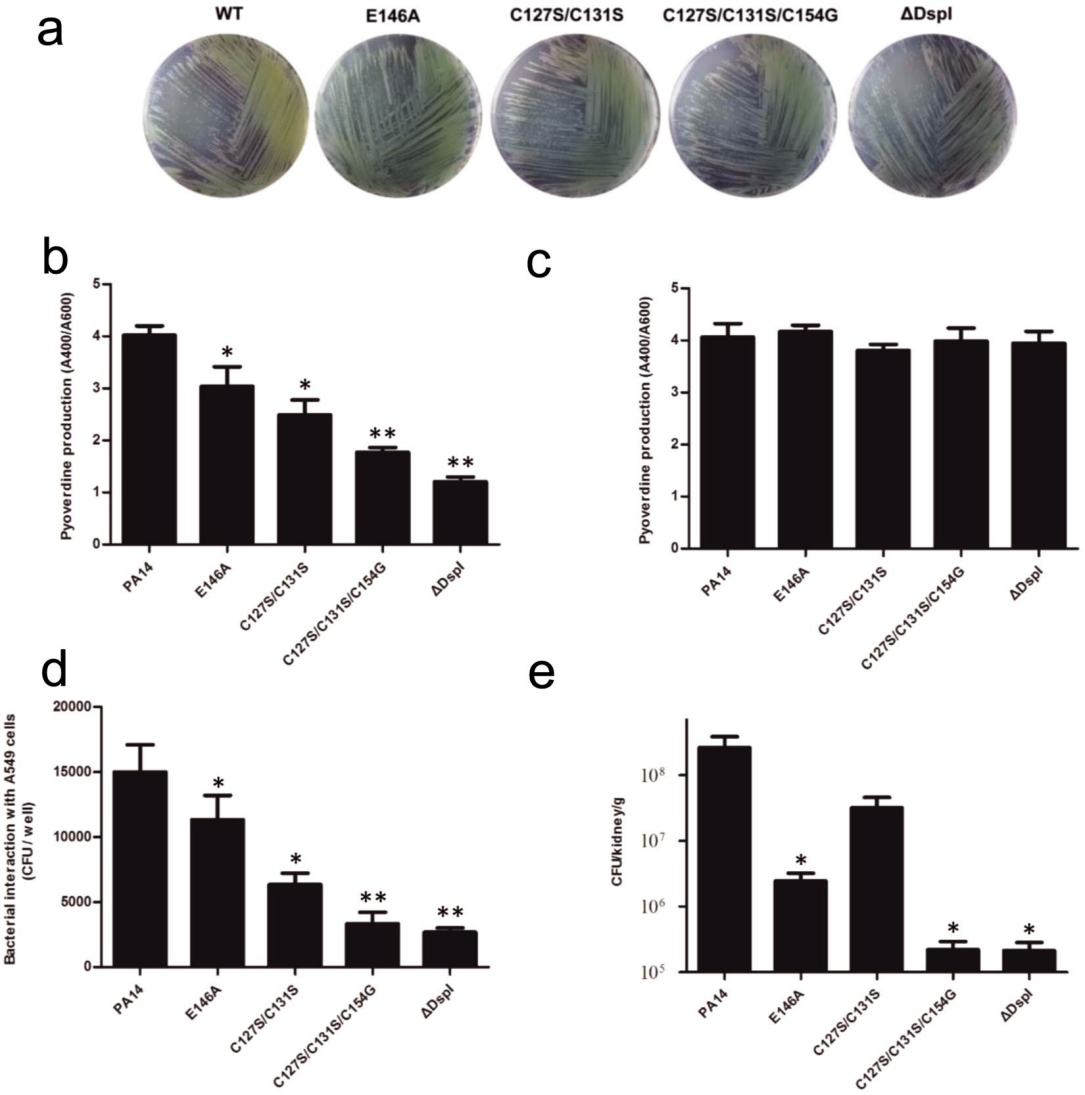
Mutations of DspI in *P*. *aeruginosa* PA14 reduced bacterial virulence *in vitro* and dispersion *in vivo*. (**a**) Wild-type PA14 and mutant strains were plated onto PIA solid medium plates. Colonies should be visible after 16 hours. (**b**) The production of pyoverdine was measured in WT PA14, E146A, C127S/C131S, C127S/C131S/C154G, and ΔDspI mutant strains in the succinate medium. Measurements were performed three times. (**c**) The production of pyoverdine was measured in WT PA14, E146A, C127S/C131S, C127S/C131S/C154G, and ΔDspI mutant strains, with the addition of 500 nM CDA compounds in the succinate medium. (**d**) Bacterial invasion (gentamicin-surviving assay) of A549 cells upon 1 h of infection at an MOI of 10 with *P*. *aeruginosa* PA14 as well as mutant strains. Measurements were performed three times. (**e**) Mice were infected with 1 ~ 2 × 10^6^ CFU/mouse of *P*. *aeruginosa* PA14 or mutant strains embedded in agar beads. Three days later, the kidneys were harvested, homogenized, and counted respectively. Data shown are represent of three independent experiments. *P* values for comparison of two groups were determined by 2-tailed Student’s *t* test (*P < 0.05, **P < 0.01 vs wild-type PA14).

*P*. *aeruginosa* is an opportunistic pathogen that causes severe infection not only in cystic fibrosis (CF) patients but also in immunocompromised patients. It produces a major siderophore pyoverdine and is found to be important for bacterial virulence and biofilm development^33^. To better elucidate the effects of *dspI* deletion on the pyoverdine production, the levels of pyoverdine (λ = 400 nm) were detected for both PA14 *ΔdspI* and the WT strains using UV-visible spectrophotometry, together with the optical density of the cultures (λ = 600 nm). The level of pyoverdine production is monitored and calculated as the relative absorption units (RU, A_400_/A_600_) (Fig. 5a,b). The results showed that the *dspI* deletion strains dramatically reduced the pyoverdine synthesis by approximately 70% compared with the WT strains and could be recovered by adding 500 nM CDA compounds in the succinate medium (Fig. 5c), which implicated DspI in *P*. *aeruginosa* virulence.

Then, we conducted a gentamicin survival assay using human alveolar basal epithelial A549 cell lines to elucidate the effects of the DspI on *P*. *aeruginosa* infection. The cells were incubated for 1 h with log phase *P*. *aeruginosa* strains at a multiplicity of infection (MOI) of 10. As Fig. 5d shows, fewer PA14 *ΔdspI* bacteria (2.67 ± 0.68 × 10^3^ CFU/well) were internalized in A549 cells, compared to the WT (1.5 ± 0.4 × 10^4^ CFU/well). Obvious attenuation effects were observed in PA14 *dspI (C127S/C131S)*, and PA14 *dspI (C127S/C131S/C154G)* strains, whereas the PA14 *dspI (E146A)* strains also demonstrated lower infection level. The results confirm that the critical roles of DspI in *P*. *aeruginosa* pathogenicity. Furthermore, we investigated the *P*. *aeruginosa* dispersion from the lung to the kidney through a chronic lung infection in mouse model^34^. As shown in Fig. 5e, in contrast to the WT strains, the PA14 *ΔdspI* strains showed reduced bacteria in the kidney on the third day after infection. Our results suggest that bacterial dispersions of PA14 *ΔdspI* are drastically weaker than that of the WT but the total bacterial infection in the lung was almost unchanged (Fig. S5). These studies further uncover that *dspI* modulates the *P*. *aeruginosa* dispersion rate by controlling the synthesis of CDA.

Furthermore, to validate the structural analysis, we generated mutations in *dspI* (*E126A*, *E146A*, *C127S/C131S* and *C127S/C131S/C154G*) to perform the functional assays (Fig. 4a). First, compared with the largely reduced swarming pattern of the E146A mutation, the E126A mutant abolished the swarming motility as well as the *dspI* knock-out strains, indicating that the role of Glu126 is more vital than Glu146. Although C127S/C131S merely slowed the dendritic growth, the triple mutant C131S/C127S/C154G remarkably decreased the swarming motility, supporting their important roles in DspI function. Further support of this notion comes from the chronic lung infection model in mice, where E146A, C127S/C131S and C127S/C131S/C154G illustrated decreased *in vivo* bacterial dispersion, which was correlated to the swarming motility results (Fig. 5e). Consistent with the structural analysis, those three mutants also presented reduced levels of either pyoverdine production or bacterial internalization in A549 cells (Fig. 5b,e).

## Discussion

The fatty acid messenger CDA produced by *P*. *aeruginosa* is a new member of the DSF family, mediating inter- and intra-species communication. It contributes to bacterial virulence, biofilm dispersion and antibiotic tolerance^11,35,36^. The increasing appreciation of its great potential clinical applications raises the requirement for understanding CDA synthesis and the signal transduction mechanism^7,37–39^. The gene *dspI* was firstly identified as an important virulence factor in *P*. *aeruginosa*^22^ and further characterized the gene coding for the key DspI enzyme for CDA biosynthesis^10^. In this work, the swarming pattern of *dspI* mutants was recovered by supplying additional CDA in the media (Fig. S2). Additionally, exogenous addition of CDA in WT resulted in enhanced swarming motility, suggesting the positive effect of CDA on *P*. *aeruginosa* swarming motility. These results confirmed the key role of DspI in pathogenicity and virulence via the CDA biosynthesis. According to the infection assay, we demonstrated that impaired flagella-dependent swarming motility caused by *dspI* mutations are correlated with weakened bacterial infection. The results verify the enzymatic function of DspI in CDA production and its role in *P*. *aeruginosa* virulence.

Pyocyanin is one of the most important virulence factor in *P*. *aeruginosa*. As a redox-active phenazine compound, pyocyanin is a reactive oxygen intermediate producer and consequently killer of mammalian and bacterial cells^33,40^. Pyoverdine is also required for establishing infection and biofilm formation^41^. In this study, deletion of *dspI* in *P*. *aeruginosa* PA14 dramatically decreased the pyoverdine production, as well as reduced the pathogenicity in the gentamicin survival assay and abolished bacterial dispersion in the chronic lung infection model. The results uncovered that DspI could be an important regulator of virulence factors expression. It has been shown that CDA-signaling involves in more than 15 cellular processes in *P*. *aeruginosa*^42^. Our study provides a valuable model for investigating the CDA-dependent signal pathway, especially the biofilm-related virulence and antimicrobial drug resistance.

DspI, as a member of the CS family, demonstrates a common crotonase motif. CS members catalyze diverse metabolic reactions with CoA-ester substrates^30^. Previous studies have classified 20 CS reactions including alkene hydration/isomerization, aryl-halide dehalogenation, (de) carboxylation, CoA ester and peptide hydrolysis *etc*., and there are three types of reactions that are involved in the fatty acid β-oxidation pathway: enoyl-CoA hydration, *Δ*^3^,*Δ*^2^-enoyl-CoA isomerization and dienoyl-CoA isomerization^23^. As a critical enzyme responsible for the 2,3 double bond formation during CDA biosynthesis, DspI has been predicted as an ECH according to its sequence alignment with the rat mitochondrial enoyl-CoA hydratase^10^. Considering the fact that ECH is an extremely efficient enzyme catalyzing the addition of water to unsaturated enoyl-CoA thioesters, while CDA production requires the reverse reaction to occur^10,43^, characterization of the structural basis holds the key to understanding the dehydration reaction mechanism of DspI (Fig. S6). The structural overlay of the ligand bound EchA8 onto the active site of the DspI allowed us to compare the reaction centers of the enoyl-CoA hydratase and dehydratase (Fig. 3d). Most of the residues engaged in catalysis are superimposed very well, namely, the two backbone amide NH groups from Ala78 and Gly123 in DspI, which play roles in stabilizing the enolates and tetrahedral intermediates during the reaction, as well as most of the CS enzymes. In EchA8 (PDB code: 3Q0G), the hydration reaction requires Glu111 to activate a water molecule for nucleophilic attack at C3 and Glu131 to protonate the C2 of the substrate. In contrast, the Glu146 in DspI is more flexible because of the missing contact between its structural equivalent Glu131 and Gln129 in EchA8. Additionally, its side chain occupies a proper position to deprotonate the C2. Furthermore, the N-terminal “helix-dipole effect” of Glu126 might be neutralized by the interaction between Cys127 and Cys131 on α3, leaving Glu126, which serves as a weaker acid to protonate the β-hydroxyl group of the substrate. Those observations from the active site comparison are applicable to propose a general acid/base mechanism of dehydration in the CS family. Additionally, the unique residue Cys154 near the active center may also contribute to this process by stabilizing and removing the later formed water molecule, implicating its critical role in catalyzing mechanisms that distinguish DspI from other ECH/ECI enzymes (Fig. 2a). Even though the schematic of the DspI dehydration steps presented here form a hypothetical picture and further research efforts are needed to verify the details, this level of investigation reveals the catalytic characteristics of the critical residues in DspI. The structural information provides a valuable template for structure-based drug development by targeting the biofilm-related infection as a complementary or alternative *P*. *aeruginosa* treatment.

## Methods

### Protein expression and purification

The full-length *dspI* gene was amplified from the *P*. *aeruginosa* PA14 genomic DNA by polymerase chain reaction (PCR) using gene-specific primers (Table S1). The coding region of *dspI* was inserted into plasmid pET-22b (+) containing six C-terminal histidine residues (LEHHHHHH) using a ClonExpressTM II One Step Cloning Kit (Vazyme Biotech Co., Ltd, Nangjing). The pET22b-*dspI* was transformed into *E*. *coli* strain BL21 (DE3) for protein expression. The bacterial culture was grown in Luria–Bertani (LB) medium in the presence of 100 μg mL^−1^ ampicillin and incubated with shaking at 37 °C until the optical cell densities at 600 nm (OD600) reached 0.9 in low speed orbital shakers (ZhiChu, Shanghai). The culture was cooled to 18 °C before isopropyl β-D-1-thiogalactopyranoside (IPTG, 0.2 mM) induction for 20 h^44^. After harvest, the cell was resuspended in a lysis buffer consisting of 25 mM Tris–HCl pH 8.0, 150 mM NaCl, 5% glycerol, and the *E*. *coli* cells were lysed by sonication. The lysate was cleared by centrifugation at 11,000 × g for 45 min at 4 °C and then the supernatant was loaded onto a 2 mL Ni^2+^-NTA affinity resin (Qiagen). The Ni^2+^-NTA column was washed with ten column volumes of lysis buffer supplemented with 50 mM imidazole. The target protein was eluted with the same buffer in the presence of 200 mM imidazole. Fractions were pooled and determined by sodium dodecyl sulfate−polyacrylamide gel electrophoresis (SDS–PAGE), followed by further purification on size-exclusion chromatography Superdex 200 column (GE Healthcare), which were pre-equilibrated with a solution consisting of 25 mM Tris–HCl pH 8.0 and 150 mM NaCl. Fractions containing DspI were pooled and concentrated to approximately 20 mg mL^−1^ using a centricon filter (10 kDa cutoff; Millipore, Billerica).

### Crystallization and structural determination

Crystallization screens were conducted as previously described with some modifications^45^. Briefly, initial crystallization screens were carried out using a Mosquito liquid dispenser by the hanging-drop vapor-diffusion method at 18 °C, using a mixing protein solution with the same volume of reservoir buffer in 96-well plates with Wizard I and II (Emerald Bio), Crystal Screen^TM^ and Crystal Screen 2 (Hampton Research), Index^TM^ (Hampton Research), and BioXtal^TM^ (Xtal Quest). The crystallization experiments were set up by equilibrating a mixture consisting of 200 nL protein solution (20 mg mL^−1^ protein in 25 mM Tris–HCl pH 8.0, 150 mM NaCl) and 200 nL reservoir solution. Crystallization hits occurred in several conditions, particularly condition consisting of 10–25% MPD, 0.1 M CH_3_COONa pH 5.0 or 0.9 M (NH_4_)_2_SO_4_, and 0.1 M CH_3_COONa pH 4.5. After optimization, the final crystallization conditions were 10% isopropanol, 5% MPD, 0.1 M CH_3_COONa pH 5.0 and 0.5 M (NH_4_)_2_SO_4_, and 0.1 M CH_3_COONa pH 4.5.

For the data collection, the crystals were soaked in cryo-protectant consisting of reservoir solution plus 20% MPD or 30% glycerol and were flash-frozen in liquid nitrogen. All the diffraction data were collected from a single crystal at beamline BL17U or BL-18U at the Shanghai Synchrotron Radiation (SSRF, Shanghai, China)^46^. The data sets were indexed and processed using HKL-2000^47^. The structure of DspI was determined by the molecular replacement method using the PHENIX package^48^ with the PaaF structure (PDB code: 4fzw) as the SWISS-modeling template^49^. Structure refinement was carried out with PHENIX refinement and rounds of manual model fitting in coot^50^. The data collection and refinement statistics are summarized in Table 1.

### Modeling of ligand bound DspI

Autodock 4.0^31^ was used to perform molecular docking of DspI in complex with the CDA-CoA precursor 3-hydroxydecanoyl-CoA. The water and ions were removed from the crystal structure of the P6_3_22 form, and hydrogens and KOLLMAN charges were added in AutoDockTools^31^. Twenty docking poses were retained and the low-energy docking score conformation was chosen.

### Construction of *P*. *aeruginosa dspI* mutations

To construct *P*. *aeruginosa dspI* mutant strains, a sacB-based strategy was employed^51^. PCRs were performed to amplify the target fragment sequences with upstream (800 bp) and downstream (800 bp) from the *P*. *aeruginosa* PA14 chromosomal DNA, while the suicide plasmid pEX18Gm was linearized with gene-specific primers. The two PCR products were recombinanted with ClonExpress II One Step Cloning Kit (Vazyme Biotech Co., Ltd, Nangjing), and the resulting plasmid, pEX18Gm-*dspI*, was then used to performed site-directed mutagenesis or deletion. All these primers are listed in Supplementary Table 1. These vectors were then transformed into *E*. *coli* S17–1 and mobilized into *P*. *aeruginosa* PA14 by conjugation to transfer the suicide plasmids from an *E*. *coli* donor S17–1 to the *P*. *aeruginosa* recipient PA14. Colonies were screened for nutritional selection, sucrose (10%) sensitivity, and gentamicin resistance, which typically indicates a double crossover event and thus the occurrence of gene replacement. The *dspI* gene mutant strains were further confirmed by PCR and DNA sequencing.

### Swarming motility assay

*P*. *aeruginosa* swarming was examined on modified M8 plates as previously described^32,52^ with slight modifications. Swarm agar was based on M8 minimal medium supplemented with 0.2% glucose, 0.5% casamino acids and 1 mM MgSO_4_, and solidified with 0.5% (wt/vol) agar (Difco). Thick plates (~25 mL/plate) were poured and the plates solidified at RT for 1 h. Then, 2.5 µL of log phase bacteria suspended in PBS adjusted to an OD600 of 3.0 were spotted on the center of each plate, which were then incubated at 37 °C for 16–24 h.

### Electron microscopic (EM) analysis

Bacteria examined by scanning electron microscopy (SEM)^53^ and transmission electron microscope (TEM) to confirm cell morphology and the presence of flagella, respectively, were prepared as previously described^54^. Cells were prepared from the edge cells of PA14 or the PA14-ΔDspI plate and were washed three times with PBS. Samples were then fixed with 2.5% glutaraldehyde in PBS overnight at 4 °C, dehydrated with a graded ethanol series (70%, 90% and absolute ethanol), respectively for 5 min each, then coated with 42 nm thickness gold and examined by SEM. Cells were also prepared for TEM by fixation with 2.5% glutaraldehyde in PBS overnight at 4 °C and then examined by TEM.

### Measurement of pyoverdine production

Pyoverdine production by *P*. *aeruginosa* growing in iron-limited succinate medium was assayed by measuring the absorbance of culture supernatants at 400 nm using UV-visible spectrophotometry, as described elsewhere^55,56^. A single colony of WT or mutant bacteria was inoculated into 3 mL of LB media and grown overnight for 24 h at 37 °C with agitation. The next day, 1 mL of the culture was diluted in 10 mL of succinate medium, and run in a 24 h culture at 30 °C under agitation. The following day, the culture was 10-fold diluted again in succinate and allowed them to grow for 24 h at 30 °C under agitation. Cells were pelleted by centrifugation at 11,000 × g for 3 min and the supernatant was diluted 10-fold in succinate medium. Pyoverdine content was determined by measurement of the absorption at 400 nm and normalized to the OD600.

### Bacterial internalization assays

Internalized bacteria were quantified by the gentamicin survival assay^57–59^. A549 (human alveolar basal epithelial cell lines) cell were purchased from the American Type Culture Collection (ATCC, Rockville, MD, USA). Briefly, mammalian A549 cells were grown in DMEM (Dulbecco’s Modified Eagle’s Medium) medium, containing 10% (v/v) fetal bovine serum (FBS; Gibco, Auckland, N.Z.) and 1% antibiotics (penicillin and streptomycin) in 5% CO_2_ at 37 °C. Suspension cultures of A549 cells were seeded at 2–5 × 10^5^ cells per well in 12-well tissue culture plates overnight at 37 °C. Cells were washed three times with phosphate-buffered saline (PBS; pH 7.2) and changed to antibiotic-free medium immediately before infection. Cells were infected with log phase *P*. *aeruginosa* strains at a multiplicity of infection^60^ of 10 for 1 h in 5% CO_2_ at 37 °C. The cells were washed twice with PBS and incubated for an additional 1 h in DMEM containing 150 µg/mL of gentamicin, which can kill extracellular bacteria. The monolayers of cells were washed three times with PBS and lysed with 0.5% Triton X-100 for 10–20 min, and appropriate dilutions were plated on potato infusion agar (PIA) plates to determine the number of viable intracellular bacteria.

### Experimental model of a chronic pulmonary infection in *P*. *aeruginosa*

We used the previous method with some modification to prepare agar beads, which were used to generate chronic pulmonary infection in C57BL/6 mice^60^. In brief, TSA (TSB with 1.5% noble agar) containing log phase *P*. *aeruginosa* PA14 was added to heavy mineral oil (prewarmed at 50 °C), immediately stirred for 6 min and then the mixture was cooled to 4 °C with stirring at a minimum speed for 35 min. The agar-beads-oil mixture rested at 4 °C for an additional 20 min and washed with sterile PBS six times. The beads diameter was measured ranging from 150 to 200 μm by light microscopy. Quantitative bacteriology was performed on an aliquot of homogenized bead slurry to determine the number of CFU per mL and the final number of bacteria was 2–4 × 10^7^ CFU/mL. We injected 0.05 mL of agar beads per mouse, allowing the beads to be implanted into the lung. In subsequent experiments, two mice in each group were weighted every day and then killed by a lethal dose of 10% chloral hydrate every other day. The lung and kidney were harvested and homogenized in PBS. Serial dilutions were plated onto PIA plates to determine the number of viable intracellular bacteria.

### Ethics statement

Mouse studies were performed in accordance with the National Institutes of Health guidelines using recommendations in the Guide for the Care and Use of Laboratory Animals. Procedures involving mice were reviewed and approved by the Institutional Animal Care and Use Committee of Sichuan University in China (Permit Number: 20160620).

### Data Availability

Atomic coordinates of the refined structures have been deposited in the Protein Data Bank (www.pdb.org) with the PDB code 5WYB and 5WYD.

## Electronic supplementary material

Supplementary information
