# Author Correction: Structural and functional studies on *Pseudomonas aeruginosa* DspI: implications for its role in DSF biosynthesis

**DOI:** 10.1038/s41598-018-30920-w

**Published:** 2018-08-17

**Authors:** Li Liu, Tao Li, Xing-Jun Cheng, Cui-Ting Peng, Chang-Cheng Li, Li-Hui He, Si-Min Ju, Ning-Yu Wang, Ting-Hong Ye, Mao Lian, Qing-Jie Xiao, Ying-Jie Song, Yi-Bo Zhu, Luo-Ting Yu, Zhen-Ling Wang, Rui Bao

**Affiliations:** 1 Center of Infectious Diseases, West China Hospital, Sichuan University, Chengdu, China; 2 Department of Dermatology, Southwest Medical University, affiliated hospital, Luzhou, China; 3 School of Life Science and Engineering, Southwest Jiaotong University, Chengdu, China

Correction to: *Scientific Reports* 10.1038/s41598-018-22300-1, published online 02 March 2018

The Acknowledgements section in this Article is incomplete.

“We thank Matthew R Parsek for the gifts of *P*. *aeruginosa* PA14 and pEX18Gm (Department of Microbiology, University of Washington). We thank Shanghai Synchrotron Radiation Facility (SSRF) beamline BL17U for beamtime allowance. We thank the staffs of the National Center for Protein Sciences Shanghai (NCPSS) beamlines BL-18U and BL-19U and SSRF, Shanghai, People’s Republic of China, for assistance during data collection.”

should read:

“The work was supported by National Natural Science Foundation of China (Grant Nos. 81670008 and 81501787) and National Key Research and Development Plan (Grant No. SQ2016YFJC040104). We thank Matthew R Parsek for the gifts of *P. aeruginosa* PA14 and pEX18Gm (Department of Microbiology, University of Washington). We thank Shanghai Synchrotron Radiation Facility (SSRF) beamline BL17U for beamtime allowance. We thank the staffs of the National Center for Protein Sciences Shanghai (NCPSS) beamlines BL-18U and BL-19U and SSRF, Shanghai, People’s Republic of China, for assistance during data collection.”

