# Supplementary material for: Structural and functional studies on Pseudomonas aeruginosa DspI: implications for its role in DSF biosynthesis
Source: Sci Rep. 2018 Mar 2;8:3928. doi: 10.1038/s41598-018-22300-1 (PMC5834635; doi:10.1038/s41598-018-22300-1)
Supplement: Supplementary file 1 — Supplementary information [file 41598_2018_22300_MOESM1_ESM.pdf]

Title: Structural and functional studies on *Pseudomonas aeruginosa* DspI: implications for its role in DSF biosynthesis

Li Liu[1,2], Tao Li[1], Xing-Jun Cheng[1], Cui-Ting Peng[1], Chang-Cheng Li[1], Li-Hui He[1], Si-Min Ju[1], Ning-Yu Wang[3], Ting-Hong Ye[1], Mao Lian[1], Qing-Jie Xiao[1], Ying-Jie Song[1], Yi-Bo Zhu[1], Luo-Ting Yu [1*], Zhen-Ling Wang[1*], Rui Bao[1*].

Supplementary Figure Legends

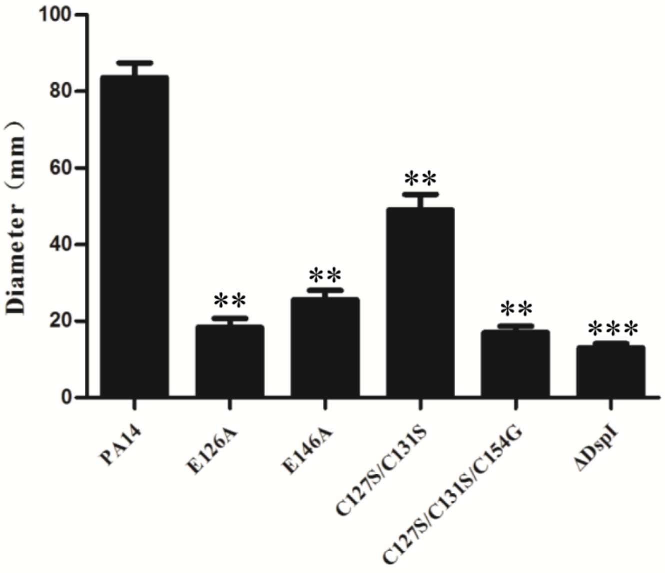

Fig. S1: Statistical results of swarming motility assays presented as the length of the colony tendril growth. Data shown are represent of three independent experiments. *P* values for comparison of two groups were determined by 2-tailed Student's *t* test (**P*<0.05, ***P*<0.01 vs wild-type PA14).

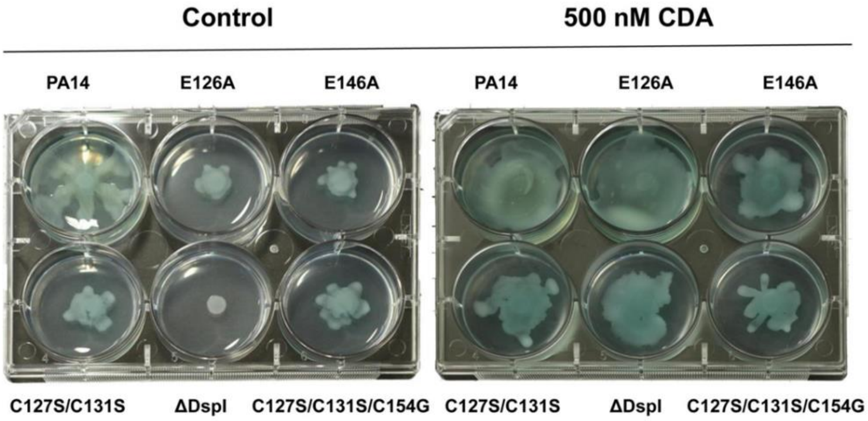

Fig. S2：The swarming-defective phenotype of PA14 mutations could be recovered after the exogenous CDA supplied. PA14 as well as strains carrying mutations in genes were incubated at 37 ℃ for 16 h in modified M8 plates supplemented with CDA at 500 nM.

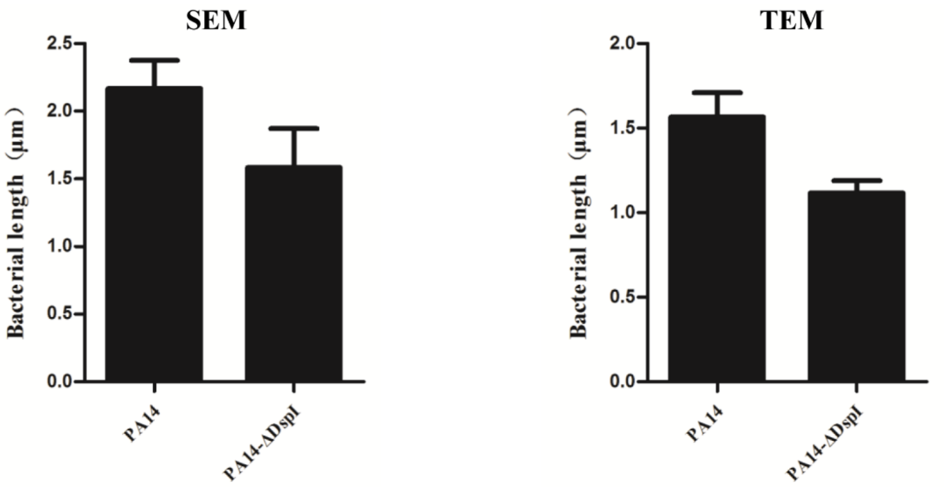

Fig. S3: Statistical results of SEM and TEM assays presented as the size of the bacteria

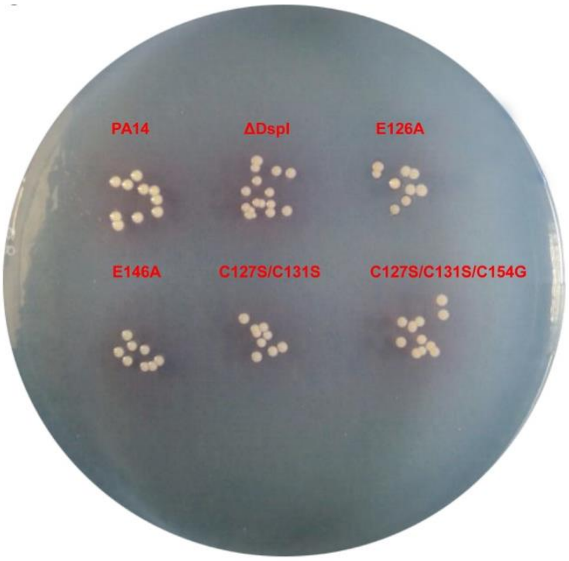

Fig. S4: The PA14 mutation strains exhibited no growth difference relative to wild-type PA14. Wild-type PA14 as well as mutation strains were serially ten-fold dilution to 10^{-6}. Dispense 10 microliters of the last dilution onto the PIA solid medium plates and then take it picture.

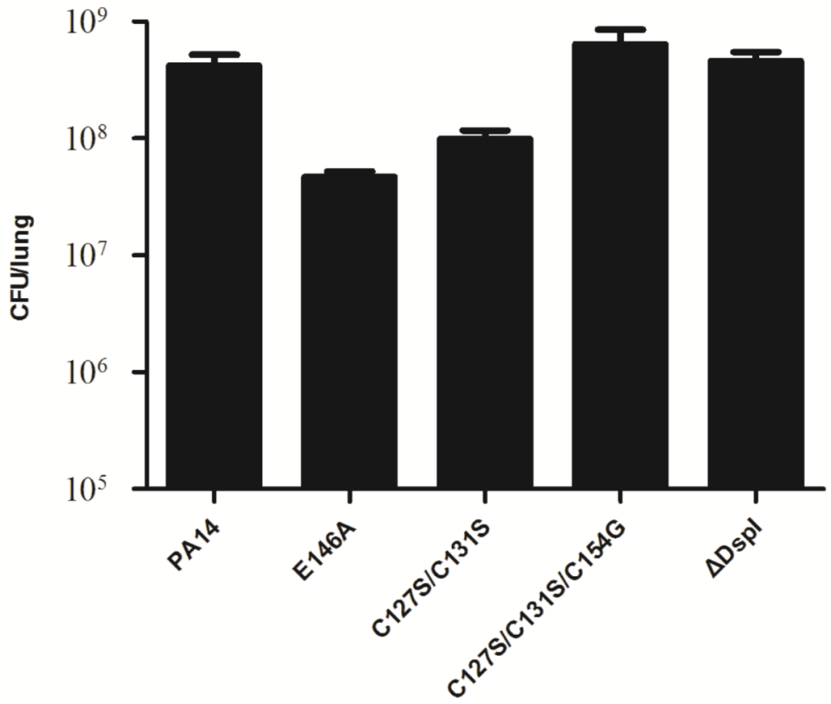

Fig. S5: Mice were infected with mutation strains in lung exhibited almost no difference relative to wild-type PA14. Mice were infected with 5~10 × 10^6 CFU/lung of PA14 or mutation strains embedded in agar beads, three days later, the lung was harvested, homogenized, and counted respectively.

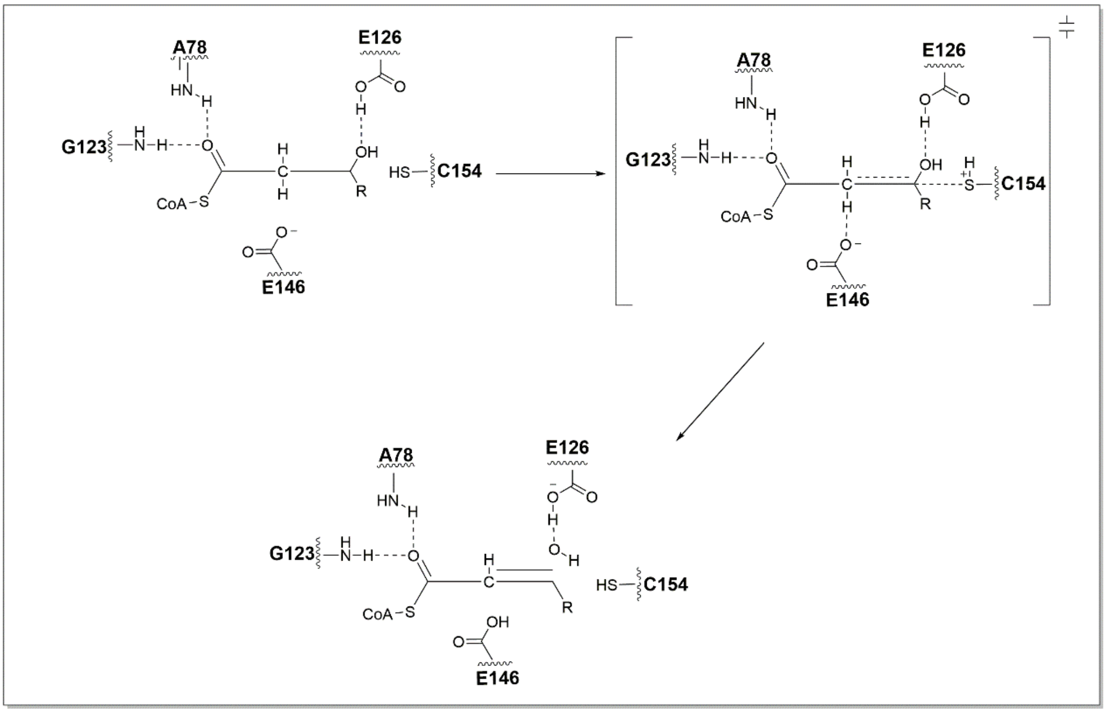

Fig. S6: Possible reaction mechanism for DspI based on its three-dimensional X-ray structure and AutoDock suite.

Table. S1. Related to experimental procedures oligo primers used in this study

Application		oligos and sequences (5’ to 3’)
Protein expression	*dspI*	pET22b- DspI-FOR: CTTTAAGAAGGAGATATACATATGATGAACACTGCCGTCGAACCC
		pET22b- DspI-REV: TCAGTGGTGGTGGTGGTGGTGCTCGAGGCAGTTGCGCCACTTGGG
	pET22b (+)	pET22b-line-FOR: ATGGTGAGCAAGGGCGAGGAGCTGTTCACC
		pET22b-line- REV: CATATGTATATCTCCTTCTTAAAGTTAAAC
Site-directed mutagenesis	PEX18Gm	PEX18Gm- DspI-FOR: AACGACGGCCAGTGCCAAGCTTGGCGACGAATACGTGCTGGACGG
		PEX18Gm- DspI- REV: AATTCGAGCTCGGTACCCGGGGATCGAGCAGTGCGTCCAGGCGCG
		PEX18Gm-line-FOR: ATGGTGAGCAAGGGCGAGGAGCTGTTCACC
		PEX18Gm-line-REV: CATATGTATATCTCCTTCTTAAAGTTAAAC
	E126A	E126A- FOR: ACGCCATGGGCGGCGGCCTGGCCTGCGCCCTCGCCTGC
		E126A- REV: AGCCGTTGATCGCCGCGATCGACACCCCGCGGAAATCGC
	E146A	E146A- FOR: ATGGCCCTGCCGGCCGCCGCGGTGGGCCTGCTGCCCTG
		E146A- REV: CTGCGCCTGGCGCTCGGCGATGCGGATGTCGCAGGCGAGG
	C127S/C131S	C127S/C131S- FOR: TGGAGTCCGCCCTCGCCTCCGACATCCGCATCGCCGAGCGCCAGG
		C127S/C131S- REV: TCGGCGATGCGGATGTCGGAGGCGAGGGCGGACTCCAGGCCGCCG
	C154G	C154G-FOR: TGCTGCCCGGGGCCGGCGGGACCCAGGCGC
		C154G-REV: TCCCGCCGGCCCCGGGCAGCAGGCCCACCG
	*Δ*DspI	ΔPA0745- FOR: GCCTTCCCATCGAACCCGGCGGGCTGCGCC
		ΔPA0745- REV: GAAGTCCCTCACTGGATCATGGGCCGGGGCGT
